# Selection methods for resistance to and tolerance of helminths in livestock[Fn FN1]

**DOI:** 10.1051/parasite/2014055

**Published:** 2014-10-29

**Authors:** Concepta McManus, Tiago do Prado Paim, Cristiano Barros de Melo, Bruno S. A. F. Brasil, Samuel R. Paiva

**Affiliations:** 1 Vice-Coordinator INCT-Pecuaria, Universidade Federal do Rio Grande do Sul, Departamento de Zootecnia Av. Bento Gonçalves CEP 91540-000 Porto Alegre Rio Grande do Sul Brazil; 2 Universidade de Brasília, Campus Darcy Ribeiro 70910-900 Asa Norte, Brasilia, Distrito Federal Brazil; 3 INCT – Pecuaria, Universidade Federal de Minas Gerais 30161-970 Belo Horizonte Brazil; 4 Instituto Federal de Educação, Ciência e Tecnologia Goiano – Campus Iporá Avenida Oeste s/n, saída para Piranhas CEP 76.200-000 Iporá Goiás Brazil; 5 EMBRAPA Agroenergia, Final W3 Norte 70770-901 Brasília Brazil; 6 Secretaria de Relações Internacionais, Embrapa, Final W5 Norte 70770-901 Brasília Brazil; 7 EMBRAPA Recursos Genéticos e Biotecnologia, Final W5 Norte 70770-901 Brasília Brazil

**Keywords:** Genome-wide selection, Selection indices, Heritability, Quantitative trait loci, Major histocompatibility complex, Animal genetic resources

## Abstract

Helminthiases are among the most important livestock diseases worldwide, in particular for small ruminants, which are the focus of this review. Resource Allocation Theory implies that high-productivity farm animals proportionate insufficient resources for adequate coping with stressful conditions. Significant differences between breeds and within breeds are seen, as well as genotype vs. environment interactions. With improvement of genetic host resistance to infection, transmission of infection will be impacted. On the other hand, genetic improvement of resilience can lead to a reduction in clinical signs of disease, but not necessarily reduce transmission of infection to other animals. Faecal egg count (FEC) is the main measurement used to evaluate helminthiasis load, despite the fact that the protocols and analytical methods can affect the results, and the FEC data frequently shows aggregative, negative skewed distribution, and a high coefficient of variation. Mass selection where heritability is generally medium to low generally produces slow results and low economic returns. Many studies have been published linking resistance to nematodes in livestock to Quantitative Trait Loci and most studies have concentrated on chromosomes where the major histocompatibility complex region is located. Nevertheless, these complex traits have been seen to be affected by thousands of variants that each has a small effect. More recent studies have shown that genome-wide selection strategies can be useful in selecting animals for improved production and resistance traits in this case.

## Abstract (fr)

Les helminthiases sont parmi les maladies les plus importantes de l’élevage dans le monde entier, en particulier pour les petits ruminants, qui font l’objet de cette synthèse. La théorie d’allocation des ressources implique que les animaux d’élevage à forte productivité répartissent des ressources insuffisantes pour faire face de manière adéquate aux conditions stressantes. Des différences significatives entre les races et au sein des races sont observées, ainsi que les interactions entre génotype et environnement. Avec l’amélioration de la résistance génétique de l’hôte à l’infection, la transmission de l’infection sera impactée. D’autre part, l’amélioration génétique de la résistance peut conduire à une réduction des signes cliniques de la maladie, mais pas nécessairement une réduction de la transmission de l’infection aux autres animaux. Le dénombrement des œufs dans les selles (FEC) est la mesure principale utilisée pour évaluer la charge des helminthiases, bien que les protocoles et les méthodes d’analyse puissent affecter les résultats, et les données FEC montrent fréquemment des distributions agrégatives de type dissymétrie négative et un coefficient de variation élevé. La sélection quantitative, où l’héritabilité est généralement moyenne à faible, apporte généralement des résultats lents et de faibles rendements économiques. Des études innombrables ont été publiées, liant la résistance contre les nématodes chez les ruminants à des loci de traits quantitatifs, et la plupart des études se sont concentrées sur les chromosomes où se trouve la région du complexe majeur d’histocompatibilité. Néanmoins, il a été observé que ces traits complexes sont affectés par des milliers de variants dont chacun a un petit effet. Des études plus récentes ont montré que des stratégies de sélection génomique peuvent être utiles dans le choix des animaux pour la production et l’amélioration des traits de résistance dans ce cas.

## Introduction

According to Perry et al. [96], helminthiases (i.e. diseases including those caused by nematode parasites) are the most important livestock diseases worldwide. Livestock selection to date has largely been based on production traits such as milk production or growth rate, with unfavourable correlated effects in fertility and health [93]. The Resource Allocation Theory [13, 54] states that animals have limited resources for carrying out adaptation processes. As production (milk or meat) is increased through one biological process, this will affect other functions such as reproduction, maintenance, movement or disease resistance. Management factors, such as increasing access to quality feed and nutrients, could increase the overall health/robustness of the animal until resources became limited again. Any further increase would imply a reallocation of resources and thus change the response in other traits such as disease resistance or behaviour [13]. Rauw et al. [101] reviewed the negative side effects of selection for high production and concluded that high productivity in livestock could mean that there are insufficient resources for adequate coping with stress factors, and hence poor welfare whenever resources are limiting.

Helminth control strategies worldwide are based almost entirely on the frequent use of dewormers (anthelmintic drugs), which are increasingly regarded as unsustainable given the emergence of multiple drug-resistant parasites [64, 129]. The need for alternative methods of control is highlighted by the fact that few new classes of anthelmintic drugs have been launched in the last 25 years (e.g. amino-acetonitrile derivatives (ADDs) and spiroindoles). Many feel that anthelmintic resistance is inevitable. Each time an anthelmintic is administered, the animal eliminates susceptible parasites and selects for resistant parasites, who then pass their resistant genes onto the next generation of worms. Interest is, therefore, growing in integrated parasite management (IPM) programmes, of which breeding for genetic resistance is a component [109].

Parasitic diseases (such as those caused by *Haemonchus contortus*, *Nematodirus*, *Fasciola hepatica*, *Dicrocoelium dendriticum*, *Eimeria* and *Amblyomma* spp. among others) are important for the sheep industry and are considered some of the biggest bottlenecks to its development [81]. Vieira et al. [127] recognised that economic losses are caused by these diseases. These include retarded growth, weight loss, reduced food consumption, decreased milk production, low fertility and, in cases of large infections, high mortality rates. The treatment of these diseases in ruminants has been affected by the emergence of nematode strains that are resistant to anthelmintics.

The acquisition and expression of immunity against gastrointestinal nematodes is genetically controlled and varies between breeds and between individuals of the same breed [81, 119]. The proportion of animals found to be resistant or susceptible to nematodes is influenced by age and breed, as shown by Amarante et al. [4] comparing Santa Inês (hair breed) with Suffolk sheep. Santa Inês has been shown to be more resistant to GI nematodes than Suffolk, Ile de France and Poll Dorset in some studies [4, 28, 30, 87] but not in others [81]. Studies show a negative correlation between the FEC and weight gain or weight of sheep of different breeds, where more resistant animals are more productive [4], although Eady et al. [46] found a positive correlation between FEC and wool production.

Genetic variation in resistance to internal parasites has been demonstrated in numerous species, including humans and several livestock species [17, 71, 99]. However, the genetic architecture underlying such traits is poorly understood [67]. The genetic aspects related to helminth control are breed, genotype-environment interactions, heritability and correlations with other traits of interest, as well as genetic markers including quantitative trait loci (QTLs) and genomic selection. Therefore, the implementation of breeding programmes becomes one of the most important factors to take into consideration as the host-parasite interaction occurs on several levels. The selection schemes can confer resistance to or tolerance of infection. “Resistance” refers to the ability of the host to resist infection, while with “tolerance” the host is infected by the pathogen, but suffers little adverse effect. For example, where the objective is to prevent the spread of the disease to other populations (as in the case of zoonotic diseases), disease resistance rather than tolerance is required.

Infection transmission is usually impacted when genetic improvement is made in host resistance to infection. While genetic improvement of tolerance may reduce clinical signs of disease, it may not reduce transmission of infection to other animals [51]. This review looks to investigate the use of genetic selection methods to improve animal response to challenges from helminths.

## Defining what trait to measure

### Direct markers

An issue that is often debated by the scientific community is whether it is more appropriate to select for resistance or tolerance (resilience). If these are separated, then improvement of the two traits could have markedly different impacts. For example, improving resistance should also reduce the transmission of infection between animals, whereas improving tolerance will reduce clinical signs of disease but may not necessarily reduce the transmission of infection. In the former case, unselected animals introduced into the population would benefit from the improvement of the herd-level resistance, whereas in the latter case unselected animals in the same environment would be at risk from disease [73].

Although resilience is usually thought of as the ability of an animal to maintain performance in the face of parasitic challenge [20], it has also been defined in terms of anthelmintic treatment requirements and anaemia following *H. contortus* infection [9]. Long-term selection for decreased treatment requirements has been shown to be successful and was accompanied by an increase in growth rate and a decrease in breech soiling [89]; however, it was complex to implement under practical conditions and did not improve resistance. Conversely, selection for a combination of resistance and performance should encompass the concept of resilience, as well as include epidemiological benefits of selection. The choice of the optimal trait to select on will often depend on the feasibility of trait recording under practical conditions [20].

Genes controlling resistance *per se* and those influencing performance are generally not associated. Consequently, genetic relationships between resistance and performance may be thought of as the outcome of a balance between two opposing factors [13, 54]: the resources used by the host to fight or protect against infection vs. the damage caused by infection. If the resources used to protect the host outweigh the benefits of being more resistant, then the relationship will be unfavourable. If the benefits of being resistant, that is, the damage that is avoided, outweigh the costs of achieving resistance, then the relationship will be favourable [20].

This framework also predicts genotype-by-environment interactions; for example, the ranking of animals on their performance may differ in environments with different challenge levels. This last prediction has been demonstrated in the comparison of Red Maasai (resistant to and tolerant of nematode infections) vs. Dorper sheep (susceptible) [8]. In this comparison, breed differences in performance that were present in the face of strong nematode challenge (favouring the resistant Red Maasai) disappeared in an environment with a low-level challenge.

Several traits are used to determine resistance or resilience to helminths. Most within-breed studies of genetic resistance use the FEC as the indicator trait for resistance, and significant heritabilities are invariably found, coupled with extensive between-animal variation in FEC [17]. The heritability of the FEC as a measure of resistance varies considerably depending on both the nematode species and breed surveyed. Estimates are generally moderate, ranging between 0.2 and 0.3 [17]; Safari et al. [106] estimated, from 16 published studies, a weighted mean heritability of 0.27 ± 0.02, with a coefficient for phenotypic variation of 31 ± 7%. Importantly, resistance to different strongyle parasites is seen to be strongly genetically correlated, and even between *Strongyle* and *Nematodirus* FEC, genetic correlations are at least 0.5 [18].

According to Bishop [21], FEC should not be the only trait considered when selecting for resistance. There are several alternative or additional indicator traits, such as: (i) measures of resistance: FEC, worm burden, worm size and fecundity; (ii) immune response: eosinophilia, and antibodies such as IgA, IgG and IgM; (iii) measures of impact of infection: anaemia, gastrin, pepsinogen or fructosamine concentrations; and (iv) resilience: growth rate and required treatment frequency.

For clinical diagnosis, the visual signs combined with the history of the animals are usually sufficient and a laboratory confirmation is not required [48]. Faecal egg counts are not suitable for confirmation of the clinical diagnosis, as correlation between the FEC and adult infection levels is usually low, but are frequently used for the diagnosis of haemonchosis in small ruminants and the detection of anthelmintic resistance (Table 1). According to the authors, the value of DNA-based tests of faecal material is therefore limited and tests of nematode species-specific DNA will have little value for diagnosis and monitoring. While pasture larval and worm counts may be useful parameters for basic epidemiological studies, they are labour-intensive, which limits their use for routine diagnosis and monitoring, as well as selection criteria. Blood parameters (gastrin, pepsinogen and serology) may be considered valuable tools for diagnosis, while pepsinogen and ELISAs based on recombinant proteins show promise as parameters for herd health monitoring [48].

**Table 1.** Summary of quantitative trait loci for gastrointestinal nematode resistance in sheep.

Reference	QTL found or nearest marker	Chromosome number	Loci studied	Trait used
[12]	OARJMP29, McM130, McM357, TGLA67, OarVH130, McMA22, McM214,	1, 3, 6, 11, 12, 20	Genome-wide	FEC
[15]	BMS360, CSSM3, BM6465, CSAP39E, MCM158, RM96, CSSM37, MCM140, CMCA52, CSAP36E, CSSME76, MAF45, BM2818, FCB128	X, 1, 6, 12	251 markers – genome-wide	strongyle faecal egg count (FEC), the coccidia faecal oocyst count (FOC) and a count of keds (Melophagus ovinus)
[23]	None	20	MHC	FEC
[25]	None	20	MHC	faecal scouring
[33]	OMHC1, OLADRB1, OLADRB2	20	OMHC1, OLADRB1, OLADRB2	faecal egg count (FEC), blood packed cell volume (PCV), antibody (AB) levels, serum proteins (SP) and blood eosinophil count (EOS)
[37]	o(IFN)-*γ*	3	o(IFN)-*γ*	FEC, IgA
[38]	BM81124, BM3215, BM3215, BM9202, ILSTS65, ILSTS42	8, 11, 23	Genome-wide	FEC, total serum IgE, serum IgGspecific for the T. colubriformis L3 larvae
[40]	IFNG, MHC	2, 3, 14, 20	139 Microsatellite markers on Chr 1, 2, 3, 5, 14, 18, 20, 21	IgA, FEC
[43]	EPCDV010, ILSTS044	1	18 microsatellite markers	FEC, Nr adult larvae
[57]	OMCH1	20	MHC Class I	-
[58]	OLADRB1	20	MHC Class II	-
[62]	None	20	MHC Class II	FEC
[63]	OarCP73, DYMS1, BM1815	20	MHC	haemotocrit level, FEC, igL
[65]	Ovar-DQA1	20	MHC Class II	Transcription profiles
[77]	Between AC113228 & ILSTS62 *and* CP26 & BMS648; *between markers* URB060 & MCMA13; *between markers* ILSTS28 & ILSTS45; *between markers* DU363924 & BMS2572 *and* MCM37 & MCM137	1, 3, 4	223 markers in Genome scan	FEC
[85]	16 genomic regions including CD53, CHI3L2, CHIA, DENND2D, RELN, NSUN2, HRH1	1, 4, 16, 19	*Genome-wide*	Selection signatures
[88]	Not specified	5, 12, 13, 21	50 K SNP chip	FEC
[94]	OLADRB	3	MHC	FEC
[95]	IFNG region BL4; BMS1617	3	fine mapping	FEC
[97]	INRA132 CP101	20	BM1818, OarCP73, OarHH56, DYA, OLADRB, CP101, OMHC1, DQA2, DQA1, TFAP2A, DQBA27, Bf94_1, and INRA132	FEC
[102]	IRF3, TGF-B1 among others	4, 12, 14, 19, 20, as well as OAR1, 3, 4, 5, 7, 12, 19, 20 and 24 at suggestive level	Meta-analysis – Illumina OvineSNP50 BeadChip	FEC
[107]	s39968, OAR12_62301297, OAR12_62347621, OAR12_62371899	7, 9, 14, 15, 21, 4, 5, 6, 8, 12, 17, 18, 19, 23, 21, 13	160 microsatellite markers were used as well as the Illumina OvineSNP50 BeadChip	FEC, PCV, worm burden, length of females, IgG, and pepsinogen concentration
[111]	*MHC-DRB1*	20	*MHC-DRB1*	*FEC*
[119]	*MHC-DRB1*	20	*MHC-DRB1*	FEC, lymphocyte antigen

### Indirect markers

Despite its advantages, the FEC requires time to measure, and may fail to represent all of the pathways involved in internal nematode resistance due to physiological complexity [45]. Increased levels of IgG1, IgE and IgM have been negatively correlated with FEC in Romney selection line sheep [22, 45, 112], although IgE was also negatively correlated with breech soiling. Immunoglobulin IgA, the isotype closely associated with intestinal mucosal immune responses, has also been positively associated with resistance [78, 114].

IgA, acting as an antibody to a *T. colubriformis* L3 carbohydrate surface antigen (CarLA), can prevent larvae from establishing in the gut, resulting in rapid expulsion [113]. A commercial test (http://www.carlasalivatest.com) was subsequently developed to measure saliva IgA antibody response to CarLa from animals under parasite challenge; high CARLA™ animals have a lower FEC, and improved growth under challenge.

An important concept is that other disease-control measures also have an antagonistic effect on genetic resistance to disease as they allow the use of otherwise unfit animals in the breeding population by preventing natural selection to disease. Animal diseases significantly decrease profitability, and identification of the phenotype for disease resistance is difficult. In a population containing both healthy and sick animals, all healthy animals may not be disease-resistant. Animals that appear healthy may have sub-clinical infections. These could then be considered pathogen reservoirs. The level of exposure of susceptible animals may also not have been sufficient to cause illness. The clinical expression of a disease can also be confounded with a similar disease; for example, pneumonia can be confused with several other diseases; bronchitis, emphysema, pleuritis, pulmonary adenomatosis, upper respiratory infection and pleural fibrosis [116]. Accurate disease diagnosis is therefore both costly and time-consuming.

The FAMACHA^©^ method was introduced to support parasite control using target selective treatment [125], based on the principle of the correlation between eye mucous colour and the haematocrit values (level of anaemia). Riley and Van Wyk [103] proposed evaluations of a practical, relatively easily obtained phenotype such as the FAMACHA^©^ score combined with simple penalties may permit rapid within-flock genetic evaluations. These could offer producers the ability to select from candidate sires and dams using ranked predicted breeding values for FAMACHA^©^ scores to improve internal parasite resistance and/or resilience, with the ultimate objective of having sheep that are able to live and produce better under conditions of relatively severe internal parasite challenge. The fact that an animal survives and reproduces without deworming can be used as a selection criterion.

## Differences between breeds

Genetic differences between and within breeds for the FEC have been seen [81]. Most studies look at differences comparing locally adapted and commercial breeds, with results showing that the relatively unselected locally adapted breeds are more resistant to/tolerant of worm infections. This may be due to poor productivity of the locally adapted breeds, as their growth rate is frequently lower than commercial breeds. This lower genetic potential for growth affects resilience, as less demand is put on nutritional partition of factors which affect both growth and resilience such as protein level in the feed [76]. In a study in the Federal District, Brazil, McManus et al. [84], in a study with several breeds and crosses, found that locally adapted Morada Nova and Bergamasca showed the lowest FEC for Strongylida, while the locally adapted Santa Inês and its cross with Ile de France (Ile X SI) had the lowest values for *Strongyloides*. On the other hand, the lowest faecal oocyst count (FOC – *Eimeria* spp.) was found in Ile de France sheep. Genetic correlations between the FEC or FOC and parasite species in these sheep were low, and heritabilities varied from 0.09 to 0.31. Nieto et al. [91] estimated heritability for the FEC using a threshold model as 0.08, but in this study animals found to have higher FECs were dewormed, which may have affected this result. Lôbo et al. [75] found FEC heritability to be highly variable throughout the life of the animal, ranging from 0.04 to 0.27 in the first challenge and 0.01 to 0.52 during the second.

The maintenance of diversity in terms of the genes underlying resistance provides an important resource for combating the effects of possible future pathogen evolution. There is much anecdotal evidence pointing to the greater disease resistance of indigenous livestock breeds to environments where they face a heavy disease challenge. A study conducted under field conditions in subhumid coastal areas of Kenya found that lambs of the Red Maasai breed showed lower FECs for *Haemonchus contortus* and lower mortality than Dorper lambs (another breed widely kept in Kenya). The Red Maasai flocks were estimated to be two to three times as productive as the Dorper animals under these subhumid conditions favourable to the parasites [10]. Likewise, Indonesian Thin-Tailed sheep have been found to show greater resistance than sheep of the St. Croix and Merino breeds [104].

Selection and crossbreeding may affect breed resistance. In Brazil, although the locally adapted Santa Inês are frequently cited as being resistant to endoparasites, usually in comparisons with commercial breeds [4, 30, 105], this was not confirmed in other studies [81]. Bricarello et al. [28] also found no significant differences between Ile de France and Santa Inês lambs in terms of their haematological and biochemical profiles under mild *H. contortus* infection. Genetic studies [82] have shown a division in this breed where crossbred animals are registered as purebred, and this may account for the lack of resistance in this “new” Santa Inês.

When resistant and susceptible breeds are crossed, studies have shown that the degree of resistance of the crossbred offspring varies depending on the breeds evaluated, the age of the animals and whether the evaluations were from natural or artificial infections [5]. Within-breed selection for the FEC has been shown to be an effective means of reducing the need for treatment with anthelmintics and reducing the contamination of pastures with the eggs of nematode parasites [18, 90, 130, 131]. Managing genetic resources in order to enhance the resistance or resilience found in livestock populations offers an additional tool for disease control.

## Selection

Few studies calculate economic values for resistance to gastrointestinal parasites [52, 74, 83]. The FAO [49] stated a number of advantages of incorporating genetic elements in disease management strategies, including: (i) permanence of genetic change once it is established; (ii) consistency of the effect; (iii) absence of the need for purchased inputs once the effect is established; (iv) effectiveness of other methods is prolonged as there is less pressure for the emergence of resistance; (v) possibility of broad-spectrum effects (increasing resistance to more than one disease); (vi) possibility of having less impact on the evolution of macroparasites such as helminths, compared with other strategies such as chemotherapy or vaccination; and (vii) adding to the diversity of disease management strategies.

Depending on the nature of the problem and the resources available, genetic management of the disease may be undertaken in a number of ways. These include appropriate breed selection for the production environment; cross-breeding to introduce desirable genes into breeds that are otherwise well adapted; and the selection of individuals that have been seen to be disease-resistant/tolerant. Genetic diversity is a fundamental requirement in all cases and populations that are diverse in terms of the number of distinct genotypes conferring disease resistance are less susceptible to catastrophic disease epidemics [117].

Selective breeding to take advantage of within-breed variation in disease resistance is an important strategy in the control of a number of diseases. Selection for resistance alone can result in negative traits such as lower live weight gains, increased breech soiling and decreased fleece weight [116]. Appropriate selection policies are therefore necessary, but the main indirect benefit of resistance is reduced pasture contamination. According to these authors, selecting for resilient animals is difficult in a commercial farming situation, although it results in animals with higher productivity, fewer dags and lower drench requirements. The major issue with resilience is pasture contamination; as there is no reduction in FEC, non-resilient animals in the flock receive a higher parasite challenge from the resilient animals, who are essentially asymptomatic carriers.

There has been considerable debate on the merits of the two approaches. Genetic correlations between resistance and resilience as high as 0.56 have been seen [3] and a heritability of resistance (0.3) significantly higher than resilience (not detectable). Bisset and Morris [22] also found low/moderate heritability (0.14 ± 0.03 to 0.34 ± 0.07), and no correlation with the FEC. Genotype × Environment interactions are thought to be low compared with the genetic factors. Kemper et al. [67] found that when *H. contortus* and *T. colubriformis* were exposed to genetically resistant or susceptible sheep over a long period of time (30 nematode generations) they did not adapt, supporting the hypothesis that resistance is determined by many genes, each with relatively small effect. This result also supports the use of selection flocks, as selection for parasite resistance based on the FEC is sustainable in the medium to long term.

It is thought that host genetic resistance may break down over time, as nematodes evolve to adapt to the resistant hosts. According to Bishop [21], the polygenic nature of parasite resistance suggests that worm evolution should be slower than that of anthelmintic resistance, as worms would have to evolve against many more targets. This author also affirms that there is no published evidence for apparently resistant breeds losing their relative advantage compared with those that are more susceptible.

Kemper et al. [68] explored three postulated mechanisms for the sheep genotype to influence the FEC in sheep: (i) reduce worm establishment, (ii) increase adult worm mortality and (iii) reduce adult egg production. These authors found that when the sheep resistance acts by reducing the adult egg production, it puts less selective pressure on the worm population, as the genes affect only the female worms and not the male. Thus, the magnitude of increase for resistant worms is only half of that when the resistance genes act on both sexes. These same authors concluded that adaptation of worms to sheep that are selected for low worm egg count (WEC) is unlikely to be detected in the short term. The authors state that the proper worm may have mutations with properties that are suitable for adaptation, i.e. mutations that are favourable for survival in low WEC sheep but unfavourable in unselected sheep. Many generations may be needed for these mutations to increase in frequency or they may be neutral (or near neutral) with respect to overall worm survival in the current population. Despite the issues, studies based on artificial conditions can be tested against natural populations, potentially providing independent validation of the results obtained. Therefore, multiple evolutionary solutions to the same problem can be tested.

According to Gibson and Bishop [51], when making decisions on the priorities for genetic improvement, the following should be taken into account: genetic improvement is an effective, low-risk method of control for the target disease; sufficient genetic variation exists for disease resistance between or within breeds to allow effective genetic improvement; there are clear economic and social benefits due to the genetic improvement of resistance, allowing for the use of other methods of disease control (used as an alternative to, or in conjunction with, host resistance).

Direct selection where heritabilities are generally medium to low which brings about slow results to selection for these traits and economic values tend to be low [74, 80, 83], but are highly affected by the price of detection and treatment.

## Genotype × Environment interaction

Animals tend to adapt to the environment they are selected in, so it is unlikely that selection for increased production levels leads to environmental sensitivity. Castillo-Juarez et al. [34] and Kearney et al. [66] showed that unfavourable genetic correlations of milk yield with somatic cell score and conception rate were significantly higher in a poor environment relative to a good environment. McManus et al. [84] showed that breeds respond differently on different pasture types, related to feed quality and availability, with differing results for FECs. Vaminisetti et al. [126] also found that breed differences were more apparent when infection levels were higher.

According to Scholtz et al. [110], due to expected changes in environments with global warming and climate change, the matching of the genotype to the environment will be important to ensure a sustainable increase in production. According to these authors, this will also be important in defining breeding objectives and to develop selection criteria that ensure that breeding is effective and aimed at sustainable production in changing environments. Definition of breeding objectives and criteria is commonly lacking in breeding programmes, especially in developing countries where the impact of environmental change is expected to be higher. In these countries, the aim of achieving maximum production may not be feasible or recommended, which is in contrast to livestock production systems in developed countries located in the northern hemisphere temperate zone. Optimal production systems are those that are in harmony with the environment and utilise appropriate genotypes, and these should be developed where they are not already in place. During the development of these systems, factors such as definition of breeding objectives should be considered and linked to factors of sustainability of production systems in changing environments [110].

## Genetic markers

The maintenance of local adapted breeds depends directly on their insertion into existing production systems. Important characteristics need to be identified in these breeds that may play an important role for specific market niches. With the development of molecular techniques, a great variety of tools are being used to improve the identification of desirable traits [117].

According to Kemper et al. [67], a further issue is that most association studies using molecular markers have relied on within-family linkage to detect polymorphisms. Thus, replicating results in a second family where the polymorphism is not segregating is almost impossible. Polymorphisms may not be segregating in families due to low allele frequencies in the breed, or because the polymorphism is fixed in a particular breed. Reproducing linkage results by identification of multiple linked QTLs is also difficult because of changes to the linkage phase between families. Many authors have studied the major histocompatibility complex (MHC) and the region containing the interferon gamma (IFNG) gene for resistance to worms [37, 40].

The MHC involves a series of highly polymorphic genes which are responsible for the initiation of the immune response when an animal is challenged by pathogens or parasites [14, 42, 50, 57, 108]. Its structure is relatively well conserved between different ruminant species, making it a candidate for comparative studies [11]. The MHC is divided into three regions: class 1 (telomeric), class 2 (centromeric) and class 3 (central). In ruminants there is a division of the class 2 region into two sub-regions: class 2a and class 2b [6]. Several studies have shown the existence of polymorphisms in each of these regions [57, 58, 72, 94]. The MHC is located on chromosome 20 in sheep [41] and its polymorphic portion is known as OLA (Ovine Leukocyte Antigen). The MHC is associated with a wide range of production traits in livestock, including sheep [25].

Several studies have been published linking nematode resistance to QTLs (Table 1) found on chromosomes 1 [12, 43], 2 [40], 3 [12, 37, 40, 95], 6 [12, 111], 14 [40] and 20 [40, 63, 111]. The QTLs on chromosome 20 found by these authors include DRB1, OARCP73, DYMS1 and BM1815. The indicator trait used by Janen et al. [63] was haematocrit level and not faecal egg count as in the majority of studies. Davies et al. [40] found two QTLs on chromosome 20 close to the MHC regions. In this case, they did not specify specific microsatellites but these QTLs were close to DRB1, OLARB, PMHC1 and CP73.

Schwaiger et al. [111] looked at the association between MHC II-DRB1 alleles and FEC following natural infection by *T.* (*Ostertagia*) *circumcincta* in Scottish Blackface sheep. Least-squares regression analysis indicated that substitution of the most common allele (I) by G2 would result in a 58-fold reduction in FEC in 6-month-old lambs, suggesting that the MHC plays an important role in the development of resistance. Since this initial study, other studies, using differing breeds and nematode species, have found the same association [36, 118, 128]. Variation in the microsatellite loci associated with functional MHC genes was also found to be correlated with juvenile FEC and survival in Soay sheep [94]. The unmanaged population of Soay sheep on the remote island of Hirta, St. Kilda, are a good model for nematode resistance. This population is persistently unstable, with numbers fluctuating between 600 and 1600 individuals [120]. Population crashes occur approximately every 3 years, primarily due to winter food shortages, although parasitism by nematodes is a contributing factor [121]. A further three markers within the MHC region had significant association with the haematocrit level (CP73), IgL (immunoglobulin lambda) level (DYMS1) and FEC (BM1815) following an artificial challenge of a Roehnschaf flock with *H. contortus* [63]. In Scottish Blackface sheep, microsatellite markers on chromosome 20 have been associated with non-*Nematodirus Strongyle* FEC [40].

Other studies using genetic marker approaches on various flocks have found no evidence for an effect of genes in the MHC on either *H. contortus* [23] or *T. colubriformis* resistance in sheep [62]. This may be explained by the alleles themselves not causing resistance or susceptibility *per se*, but being in linkage disequilibrium (LD) with additional polymorphisms in the region [65]; a combination of these polymorphisms may then contribute to resistance or susceptibility in some populations. As the extent of LD between those populations is likely to vary between breeds and populations, the MHC alleles previously implicated may not show up as being significant.

Quantitative trait loci (QTL) mapping has been widely used to try to understand the complexity of parasite resistance. This is carried out by identifying regions of the genome involved in variation in the phenotype. Diverse experimental approaches have been used, mainly based on comparing sheep breeds and nematode species at different ages and under different climatic/management conditions. Because of this, different chromosomal regions of interest have been suggested as being involved in this resistance/resilience. The results suggest that several different pathways are involved in nematode resistance. The region near the MHC on chromosome 20, and the region containing the IFNG gene on chromosome 3 are two regions that are frequently targeted during these studies.

The IFNG gene codes for a cytokine secreted by Th1 lymphocytes that plays a critical role in regulating the type 1 vs. type 2 immune responses in vertebrates. It activates macrophages, which can kill intracellular pathogens, and display increased ability to present antigens [129]. INFG is used as a candidate for nematode resistance as it is associated with host response following an immune challenge. It also helps to determine whether a humoral or cell-mediated response predominates.

A QTL for parasite resistance in Romney divergent selection lines after multi-species challenge was fine-mapped to a region near the IFNG gene [95]. Subsequently, polymorphism in the region near the IFNG gene was linked to reduced FEC and increased parasite-specific IgA in a wild population of Soay sheep on the island of Hirta in the St Kilda archipelago in the Outer Hebrides [37]. This region has been implicated in a number of host resistance traits, including specific IgA activity, *Nematodirus* FEC, non-*Nematodirus* and strongyle FEC. These have all been identified to be associated with a QTL on chromosome 3 in Scottish Blackface sheep [40], using a partial genome scan of 139 microsatellite markers. Likewise, using 133 markers, a QTL in the IFNG region was also observed in Merino divergent selection lines after challenge with *T. colubriformis* [12]. Finally, a whole genome scan using 247 markers was performed on the Soay sheep of St. Kilda, using the coccidian FOC as a measurement of resistance to parasites. Several other regions on chromosome 3 have also been found to have linkage to parasite resistance, although they are not found near the IFNG [15, 77].

A region at the distal end of chromosome one was also found in Merino selection lines to be significantly associated with a mean FEC of three counts after secondary artificial challenge with *T. colubriformis* [12], using an incomplete genome scan of 133 markers. A genome scan using 203 microsatellites and divergent Romney FEC selection line outcrosses naturally infected by *Trichostrongylus* species detected a significant QTL on the telomeric end of chromosome 8 [38]. A further partial association screen using 139 microsatellite markers has been used to also identify QTLs associated with *Nematodirus* FEC in Scottish Blackface sheep on chromosomes 2 and 14 [40]. These QTLs are known to affect egg production by *Nematodirus* species, although potential candidate genes have not yet been identified.

Over three successive population crashes in the Soay sheep of St. Kilda, Gulland [105] showed that mortality was significantly different among individuals of the three different genotypes at the diallelic adenosine deaminase (ADA) locus on chromosome 13 [16]. Three independent lines of evidence suggested that nematode burdens differ among the three genotypes, consistent with the idea that allele frequencies at the ADA locus are maintained by parasite-induced selection. Microsatellite work on the same breed, with 251 markers covering the whole genome, later failed to find any linkage between FOC and the ADA locus, INFG or MHC, all regions previously proposed to be candidate loci [15]. The failure to detect linkage could be due to insufficient power, or low marker coverage, although good marker coverage was achieved in the putative regions [16]. More recent studies with this population [29] have concluded that there is little evidence that the candidate gene approach will lead to the identification of loci explaining variation in parasitological and immunological traits.

The same genome scan produced a high LOD score for chromosome X, in the vicinity of one of the telomeres [15]. This was the only study to analyse the X chromosome for linkage until recently, when Marshall et al. [77] discovered a QTL in the X-chromosome pseudoautosomal region, using resistance to *H. contortus* infestation in Merino sheep. This study used 223 microsatellite markers on all autosomes, plus the X-chromosome pseudoautosomal region. The QTL on the X chromosome was discovered alongside many other QTLs, of which only three (located on chromosomes 1, 3 and 4) were fine-mapped. While the locations for the significant markers were given, it did not appear that any of these QTLs were located in known genes.

Breeding plans should be constructed to implement strategies designed to maintain genetic variability and prevent the increase of inbreeding. This can be accomplished by: (i) including health, fertility and other fitness traits in breeding objectives along with production traits; (ii) taking genotype-by-environment interactions into account; (iii) implementing selection strategies to reduce inbreeding; and (iv) taking advantage of the molecular genetics tools.

## Differences among studies

The search for QTLs for nematode resistance in sheep is a difficult area of research. This is primarily due to the physiological and phenotypic complexity of the trait, and most studies derive from initial low-resolution genome screens, often resulting in very wide confidence intervals. More consistency in experimental protocols, materials and analysis approaches would allow a more accurate comparison of results; the studies have differed in the breed of sheep and their immune status, nematode species used in the experiments, measurement of internal nematode resistance and the challenge regimes. Comparisons between breeds of sheep, such as locally adapted and commercial breeds, are also problematic for many reasons including differences in environment, age structure, treatment history and parasitological methods. Considering the complexity of nematode resistance, it is unsurprising that previous studies have not necessarily yielded the same results.

Differences in experimental design aside, due to the moderate size of the effect of each QTL, it is likely that a panel of QTLs would be required for significant genetic gains to be achieved within the industry via marker-assisted selection. The information gained from such QTL studies can be used alongside new technology to gain a greater understanding of nematode resistance in sheep; as noted by Crawford et al. [38], the large number of suggestive QTLs discovered suggests that most of the genes controlling parasite resistance are of relatively small effect.

## Genome-wide selection

Single nucleotide polymorphisms (SNPs) are DNA sequence variations that occur when a single nucleotide differs between different homologous chromosomes. Studies of variation at a single base pair level can provide information of two kinds; firstly, it can be used to study polymorphisms within protein coding regions, and secondly, variation can also be studied in non-protein coding regions, including regulatory regions. It has been suggested that much of the evolution of morphology, physiology and behaviour rests on changes in regulatory sequences, and thus they can have a profound effect [32]. Recent studies have also shown the role of non-coding RNAs, including microRNAs and nsRNAs, in regulating various levels of gene function [79].

Due to their abundance, ease of scoring and low unit cost, SNPs are now the most widely used markers in genetics, allowing the development of dense catalogues of variation within a species [123]. In the field of ovine genomics, technology such as the Illumina® OvineSNP50 or OvineSNP700 K BeadChips (www.sheephapmap.org) now enables researchers to characterise the genetic variation at more than 50,000 SNPs in the ovine genome simultaneously. This first genome-wide set of SNPs for sheep has opened the gateway for further research [69, 70].

Progress in genomics, along with technology, statistical techniques and bioinformatics advances, has led to the implementation of genome-wide association studies (GWAS) which aim to understand the genetic basis of common diseases. Infectious diseases are important on a global scale and there is strong epidemiological evidence that host genetic factors are important determinants of the interactions between host and pathogen. However, the application of GWAS to infectious diseases has been limited when compared with non-communicable diseases. Gilleard [53] has reviewed the literature on using this approach with *H. contortus* as a model.

Association studies with candidate genes, which look for a statistical correlation between specific genetic markers and a disease, have been widely used for the study of complex diseases [31], yet this approach had been criticised due to inability to replicate results and limits on its ability to include all possible causative genes and polymorphisms [122]. These limitations have been overcome by population genomics [1], where the genome, or at least a large number of loci (often well into the 10,000 s) that are likely to be representative of the whole genome, is surveyed without any prior assumptions regarding which genes are under selection, resulting in less bias.

Kemper et al. [67] used a mixed-breed population of sheep to show that the detectable polymorphisms affecting resistance to worm infections have relatively small effects. Considering that the additive genetic effects for the WEC accounted for between 10 and 24% of the phenotypic variance in this population, this means that there are likely to be hundreds or thousands of underlying mutations influencing these phenotypes. These mutations are probably spread across the genome. This is in line with Al Kalaldeh et al. [2], who concluded that disease resistance is a largely polygenic trait. This implies that there are a large number of genes involved in the mechanisms of resistance but there are some chromosomal regions that explain a larger proportion of the variation. When all markers were used, a moderate proportion of the genetic variance in the trait was explained. However, improvements are still necessary and previous research in dairy cattle shows clearly how the accuracies of genomic predictions can be increased.

The first step is to increase the size of the reference population. Deterministic predictions indicate a steady, almost linear, increase in GEBV accuracy if up to 20,000 extra records were added to the reference dataset [55]. The rate of increase is conditional on the heritability of the trait, and thus artificial challenges for the reference population may still be required to maximise exposure to infection and the potential heritability for WEC [19]. The next step is to increase the potential LD between markers and polymorphisms by increasing the density of SNP markers. Low LD between markers in breeds such as Merinos suggests that the value of increasing the size of the reference population will be limited unless the density of markers is increased.

## Problems with GWAS

While GWAS have identified hundreds of common genetic variants associated with complex disease so far, most confer relatively small increments in risk, in contrast with the initial “common disease, common variant” hypothesis [125]. The question was then raised how the remaining, so called “missing”, heritability can be explained. While it has been postulated that rare variants, epistasis, epigenetics and genotype-environment interactions might explain the missing genetic influence [47], the more likely reason is that complex traits are affected by thousands of variants that each have a small effect. The genetic variants now used in most studies were identified in a small number of presumably healthy humans/animals, whereas it may be the rare and low-frequency variants (MAF < 5%) that are in fact contributing to the “missing” heritability.

This hypothesis has been validated by Yang [131], who concluded that much of the heritability for height can be captured by common variants undetected by GWAS, due to individual effects being too small to pass the stringent significance tests. The authors also provided evidence that the remaining heritability is due to incomplete linkage disequilibrium between the SNPs genotyped and the causal variants, which would be exacerbated by the causal variants having a low minor allele frequency [128].

Greater than 70% power to detect QTLs explaining 0.1–0.48% of the phenotypic variance requires a higher level of LD between markers and polymorphism, and a greater number of observations [67]. Increased marker density or using a single breed with low effective population size, such as Poll Dorset, would increase the likely LD between markers and polymorphism. Reliable detection of polymorphism explaining about 0.5% of the phenotypic variance (e.g. 70% power), could be achieved when the LD between markers and QTLs is 0.4 and with about 10,000 records [67]. Detection of smaller polymorphisms, such as those estimated for the WEC found by this author, would require greater marker density and many more phenotypes.

## Selective sweeps

When an advantageous allele fixes in a population, it does so on a particular haplotype background. The advantageous mutations sweep through the population, along with linked variation, in a process referred to as a “selective sweep” [111]. When this occurs, it leaves a characteristic signal in patterns of variation in genomic regions linked to the selected site. In the absence of recombination, all neutral SNPs on the chromosome also become fixed, thus losing all variability in the region. New haplotypes emerge through recombination, with the effect of the selective sweep (linkage disequilibrium) diminishing with distance from the advantageous allele. Incomplete selective sweeps denote any stage prior to the fixation of the advantageous allele.

Predictions of genetic merit from all markers are being implemented in livestock breeding programmes, typically within a single breed or strain of animals [56, 60, 61, 124]. Kemper et al. [67] found that there are many polymorphisms of small effect underlying variation in FEC. These authors found the largest effects were estimated to explain between 0.12 and 0.48% of the phenotypic variance for the WEC following challenge with *T. colubriformis*, and between 0.02 and 0.08% of the phenotypic variance following *H. contortus* challenge. The additive genetic effects for the WEC accounted for between 10 and 24% of the phenotypic variance in this population; this means that there are likely to be hundreds or thousands of underlying mutations influencing these phenotypes. These mutations are probably spread across the genome.

## Looking at the worms

The relationship between the parasite and its host leads to the establishment of a co-evolutionary process where the selective pressures imposed by both players shape each other’s genome through time. Thereby, changes in gene frequencies leading to the fixation/loss of favourable/unfavourable alleles from both host and parasite populations become constant and counterbalanced, giving rise to a process that can be compared with an endless arms race [59]. Thus, the modification of genetic features of host populations during breeding programmes is expected to have an impact on the parasites’ genetic pool.

In this context, although resistance and resilience may have a similar impact on individual health and productivity, they can have contrasting effects on disease prevalence at a population level [44]. For example, breeding for resistance has the potential to decrease the parasite load in the pasture, therefore providing clear epidemiological benefits. On the other hand, breeding for resilience is not expected to enhance the pathogen-host arms race at a rate as fast as the selective pressure imposed by resistance would do. This is a theoretical prediction that is important to highlight, since nematode infections are highly prevalent and several nematode species have a broad host spectrum [21]. Together, these characteristics hinder pathogen eradication, the ultimate consequence of resistance, unlikely.

It is possible to speculate that the selective pressure imposed by anthelmintic treatment has similar effects to those the resistant host background would have upon the parasite population. Thus, it might be useful to take a look at the extensive amount of data gathered in the last decades regarding anthelmintic resistance rise and spread (reviewed by Prichard [98] and Molento et al. [86]). After years of widespread use of drugs for the control of parasitic infections, the increasing prevalence of resistant nematodes now threatens the production of livestock in several parts of the world. The continued use of anthelmintic drugs did not eradicate the parasites, mainly because the majority of the nematode populations were in *refugia* (e.g. eggs and larvae in the pasture, inside asymptomatic animals or inside animals of other host species), and therefore not submitted to drug selection. Moreover, the genetic pool present in the populations in *refugia* started to continuously supply drug resistance-conferring mutations, which eventually became fixed. The situation is further complicated by the usually low genetic structure of parasite populations and their high mutation rates [7, 24, 26, 27, 39, 92, 114]. Indeed, there are many reports describing that resistance can originate through different ways such as animal movement among farms [24, 26, 35] and novel/recurrent mutations [27, 114, 115].

When looking at the possibility of gastrointestinal worms adapting to sheep bred for a low faecal egg count, Kemper et al. [68] concluded that this requires an allele segregating in worms that is favourable in animals with improved resistance but less favourable in other animals. They state that the chance of obtaining alleles with this specific property seems unlikely and conclude that selection for a low faecal egg count should be stable over a short time frame (e.g. 20 years).

Another important feature of the anthelmintic resistance rise is that it took decades to become a major production problem. It seems that the same populations in *refugia* also functioned as buffers to the drug’s selective pressure and slowed the process of resistance rise. This hypothesis is supported by the expectation that a low-prevalence, host-restricted pathogen would have become either quickly eradicated or resistant [44].

Therefore, given the different outcomes that breeding for resistance or resilience might have on the parasite populations it would be useful and more sustainable to combine both features. Indeed, breeding for resistance might be more effective in the short term, especially given its higher heritability compared with resilience traits [21]. Furthermore, the selection of more virulent parasite populations might progress slowly, similarly to what happened to anthelmintic resistance. Nonetheless, resilience will be an important trait since it is unlikely that resistance will eventually lead to the eradication of these highly prevalent and broad-spectrum nematodes. In addition, there is no reason to believe that these traits are mutually exclusive [100], so greater performance gains should be achieved by the coupling of resistance and resilience.

## Conclusions

The co-evolution of hosts and parasites is one of the most interesting examples of the evolutionary history of organisms, yet the complexity of both parasitic infections and host immunity makes it a challenging field to study. Studies aimed at understanding the underlying genetic factors in mammals can help with our understanding of the ongoing struggle between the parasite and host for evolutionary dominance. Whereas studies in humans traditionally lack statistical power, with sheep, the ability to manipulate breeding lines, replicate studies, and have accurate recorded pedigrees offers significant advantages, including increased statistical power. It also allows us the potential to test whether there are multiple evolutionary solutions to the same problem, through having independent selection lines.

Integrated disease control strategies are indicated in the control of these parasites. These include improved management, nutrition and careful use of anthelmintics. More recently, there has been increased interest in the selection of small ruminants for improved nematode resistance to complement other control measures [20]. The existence of genetic variation in resistance, both within and between breeds, means that this may be successful. This involves the choice of appropriate breeds that are adapted to local environmental conditions, followed by phenotypic selection for resistance. Selection objective traits include performance (e.g. growth rate) under conditions of parasite challenge, FEC and measures of anaemia (Table 1).

There may be genotype-by-environment interactions, especially when animals are reared in environments that differ in the level of parasite challenge or the quality of available nutrition [74, 82]. Nevertheless, antagonistic genetic relationships between performance and resistance are not expected, and selection indices should be constructed that improve both traits. If the FEC is decreased, then pasture contamination should also decrease [20], leading to additional benefits for all sheep grazing the same pasture. These authors state that breeding for nematode resistance should lead to lasting and sustained improvements in resistance or resilience. It is thought that nematodes will not evolve rapidly in response to resistant hosts, and research suggests that resistance should be sustainable.
